# A four-year, mixed-family and community-based growth monitoring and promotion program using multi-level modeling to address undernutrition in children in Cambodia

**DOI:** 10.3389/fpubh.2025.1572941

**Published:** 2025-06-24

**Authors:** Po-Yen Liu, Yen-Ting Lin, Yee-Hsuan Chiou, Maw-Sheng Lee

**Affiliations:** ^1^Institute of Medicine, Chung Shan Medical University, Taichung, Taiwan; ^2^Royal Artemis Hospital, Kaohsiung, Taiwan; ^3^Division of Pediatric Nephrology, Department of Pediatrics, Kaohsiung Veterans General Hospital, Kaohsiung, Taiwan

**Keywords:** family based interventions, under 5 years’ nutrition, Cambodia, community based interventions, growth monitoring and promotion

## Abstract

**Introduction:**

Child undernutrition persists as a formidable public health issue in developing countries. Children afflicted by undernutrition are susceptibility to both physical and neurological repercussions. For several decades, initiatives focused on growth monitoring and promotion have been instituted to mitigate this pressing issue. Nevertheless, the prevalence rates of undernutrition across developing nations continue to provoke concern.

**Methods:**

Between the years 2016 and 2019, we executed a family- and community-oriented growth monitoring and promotion initiative within a rural Cambodian village, specifically aimed at children under the age of five. This initiative employed a hybrid workforce comprising both full-time health professionals and community volunteers. Leveraging this robust capacity, we delivered small-group nutrition education sessions, family-centered nutrition counseling, and regular anthropometric assessments. In contrast to a cross-sectional methodology, we used multi-level modeling to explore the growth trajectories of children utilizing longitudinal z scores for height-for-age and weight-for-age. A systematic taxonomy of models was developed in a sequential framework to ascertain the most appropriate final model.

**Results:**

Out of 533 enrolled children, 358 completed the growth monitoring program (GMP). At baseline, children older than 12 months had significantly lower height-for-age (HAZ) and weight-for-age (WAZ) scores, as well as higher rates of stunting and wasting, compared to younger children. These differences were no longer significant by the end of the program. Nonparametric trajectory analyses showed age-related differences in HAZ patterns, with initial declines followed by recovery in younger age groups, while WAZ trajectories remained relatively flat across all ages. Multilevel modeling indicated that both age at enrollment and time significantly influenced HAZ changes, while only age at enrollment affected WAZ. Older children exhibited steeper improvements over time, leading to convergence in growth outcomes with younger children.

**Conclusion:**

An extended and efficacious growth monitoring and promotion program has the potential to ameliorate the issue of undernutrition in developing countries.

## Introduction

Growth Faltering in children, particularly during the first 1,000 days of life, has both short-term and long-term consequences. In the short term, it increases the risk of morbidity, mortality, and impaired neural cognitive development during childhood. Over the long-term, undernutrition status can negatively impact human capital attainment and lead to economic loss (1–6). Despite years of efforts to eliminate childhood undernutrition in low- and middle-income countries, growth faltering remains a significant public health challenge (7–14). In 2020, estimated 149.2 million children under-five (22.0%) were stunted while 45.4 million (6.7%) were wasted globally (15). Given this persistent high prevalence, effective strategies are still needed to reduce the burden of undernutrition.

Growth monitoring and promotion (GMP) has been widely implemented to prevent childhood growth decline since its introduction in the 1970s (16). The World Health Organization (WHO) released a guideline in 2017 advocating GMP as a key intervention against child malnutrition (17). Regular anthropometric measurements, early detection of growth faltering, nutritional counseling/interventions are core elements of GMP. While anthropometric measurement alone does not improve health outcomes, timely and appropriate follow-up interventions are essential to mitigate growth faltering and enhance child nutrition. A well-coordinated execution of its core elements is essential for success. Currently, there is no standardized definition of which specific interventions should be included in a GMP. Additionally, the perceptions of policymakers and resource limitations influence the scale and effectiveness of GMP interventions Common challenges affecting GMP outcomes include low participation rates, weak implementation, poor coordination within child health programs, staff shortages, and inadequate training and monitoring (18–22). Despite being implemented in various developing countries for decades, the effectiveness of GMP in combating childhood undernutrition remains uncertain (18, 20–22).

Over the years, child growth standards have been updated several times to improve the accuracy of anthropometric measurements. Currently, WHO child growth standards, based on multi-country child growth pattern, serve as a representative reference for tracking of child growth curve. By using WHO growth standards, a child’s actual height and weight are converted into height-for-age z-score (HAZ) and weight-for-age z-score (WAZ), which provide objective evaluations of nutritional status. Stunting (HAZ ≥ 2 SDs below the median) indicates chronic malnutrition, while wasting (WAZ ≥ 2 SDs below the median) reflects acute nutritional deficiency. These two indicators are widely used to assess a child’s nutritional status and monitor progress in nutrition interventions. Regular anthropometric measurements allow for early detection of growth faltering, prompting follow-up interventions and nutrition counseling for caregivers to help reverse negative growth trends.

Cambodia is a low-income country with a high prevalence of childhood undernutrition (7, 23–28). Addressing this issue is an urgent public health priority the growth trajectories of Cambodian children follow patterns similar to those observed in other developing countries (29–35). To bridge the gap between GMP theory and its practical application, we conducted a prospective GMP in a Cambodian rural village, extending over 4 years, which was longer than most previous GMPs. We hypothesized that a longer GMP duration would provide a more comprehensive representation of child growth trajectories in developing countries. To ensure data accuracy and consistency, we hired four health workers in this GMP to conduct anthropometric measurements to minimize errors. Measurements were taken at multiple locations close to households to increase coverage and reduce missing data. To enhance caregiver engagement, local staff visited households periodically to provide personalized nutrition counseling. For data analysis, we used multi-level model (MLM) instead of a cross-sectional approach. This model is well-suited for longitudinal data, as it accounts for both within-person changes over time and variations between individuals, rather than merely capturing average population-level changes (36–40). Demographic factors were also incorporated into the model for further analytical insights. Our study aim was to investigate the impact of a longer mixed-family and community-based growth monitoring and promotion program on growth trajectories of children in developing countries.

## Materials and methods

This (GMP) program was implemented in Pot Sar commune, Takeo province, Cambodia, from August 2017 to December 2020 by Kaohsiung Veterans General Hospital. Pot Sar commune consists of 11 villages with a population of 13,519 as of 2019 and Takeo province is one of Cambodia’s poorer regions. Before launching this GMP, Kaohsiung Veterans General Hospital had been working with Green Umbrella, a local non-governmental organization (NGO) based in Pot Sar commune that has been involved in children’s education and healthcare since 2012. The partnership provided essential access to the relatively isolated Pot Sar community.

Children under 5 years of age residing in Pot Sar commune were eligible for participation. Children with pre-existing medical conditions, such as congenital heart diseases, sickle cell anemia, and other genetic and metabolic disorders that could interfere with normal growth patterns, were excluded from participation. Participants and local volunteers were recruited through Green Umbrella, leveraging its established community presence. After obtaining informed consent, children were enrolled in three phases: August 2017, April 2018, and October 2018. To ensure accurate data collection and effective program delivery, four local employees were hired as health workers, responsible for anthropometric measurements, educational sessions, and home visits. A full-time pediatrician from Kaohsiung Veterans General Hospital was stationed in Pot Sar commune to coordinate and supervise the health workers. Before the program began, the health workers underwent a five-day training session, covering communication skills, anthropometric measurement techniques, and nutrition education. Each health worker was provided with a laptop for secure storage of anthropometric and demographic data. To enhance community engagement, a Pot Sar Children’s Nutrition Improvement Committee was established, integrating local political leaders into the program. The committee included: the leader of Pot Sar commune, local leaders from the 11 villages, the superintendent of Pot Sar healthcare clinic, and the founder of Green Umbrella. Committee meetings were held monthly, where the pediatrician and health workers presented progress reports and discussed upcoming activities. To further strengthen community participation, 32 local volunteers were recruited across the 11 villages. These volunteers attended annual five-day nutrition training sessions throughout the four-year program. The enrolled children were divided into small groups, each consisting of 15–20 children based on geographical proximity. Each group was assigned one volunteer, who collaborated closely with health workers to coordinate anthropometric measurements and nutrition education sessions.

### Interventions

We conducted nutrition education sessions within each of the small groups to foster engagement and knowledge retention among caregivers. To boost attendance, we introduced a “loyalty stamp and punch card” system, where caregivers who met attendance thresholds received incentives in the form of nutrition supplements. We anticipated that small-group setting would create a more intimate learning environment, encouraging open discussions between caregivers and health workers. To enhance accessibility, the education sessions were held every 3 months at volunteers’ homes, ensuring proximity to caregivers’ residences. The sessions covered a comprehensive range of topics relevant to child-rearing, including introduction of three macronutrients, breastfeeding, timing of starting complementary feeding, personal hygiene including hand wash skills, environmental sanitation, food and drinking water safety, common health issues in this age group, importance of vaccination card, and procedures about anthropometric measurements. Given the high illiteracy rates in this community, we structured the sessions in an interactive game-based format to create an engaging and competitive learning atmosphere. To further reinforce key messages, brochures with pictures and illustrations were distributed to caregivers for home review. To raise awareness about the importance of nutrition, we also installed three large advertising tarps along major roads in the community to capture residents’ attention and emphasize the significance of child nutrition.

Deworming and vitamin A supplementation have been routine services provided by the Pot Sar Health Care Clinic prior to our GMP. However, the coverage rates for deworming drugs and vitamin A supplements were only 73.5 and 83%, respectively. To enhance tracking, we proposed that, in addition to immunization records, the history of deworming administration should be marked on the existing immunization cards by the Pot Sar Health Care Clinic. If no deworming record was found on a child’s vaccination card during anthropometric measurement, we administered the necessary deworming drugs. We also worked on improving local dietary practices by modifying traditional recipes to incorporate more locally grown ingredients and nutrients. First, we demonstrated these revised recipes to the 32 volunteers. Then, we introduced them at various social events, allowing caregivers to sample the new dishes. To further promote these changes, we held cooking sessions led by volunteers at these events. Additionally, we distributed pamphlets containing the modified recipes to inform all caregivers. To assist caregivers in preparing nutritious meals at home, we developed a visual aid tool featuring three illustrations representing the key macronutrients: carbohydrates, proteins, and fats. The size of each illustration corresponded to the appropriate serving portion for a balanced meal. This visual aid was printed on a waterproof plastic plate for easy use in the kitchen, helping caregivers provide well-balanced meals for their children. Finally, to reinforce proper hygiene practices, we created a handwashing song with choreography and demonstrated it at every educational session. This interactive approach kept children engaged and encouraged them to incorporate handwashing into their daily routines.

Home visits served as the foundation of our GMP intervention. Nutrition counseling was provided to all participating caregivers at the household level. Each month, health workers, accompanied by volunteers, visited caregivers to offer support and guidance. During these visits, health workers used a structured questionnaire to guide their interviews, ensuring discussions aligned with the topics covered in nutrition education sessions. They provided tailored responses to caregivers’ questions based on their specific contexts and assessed behavior changes firsthand. When necessary, health workers reinforced nutrition knowledge to help caregivers solidify their understanding. Additionally, household sanitation was evaluated during each visit, with recommendations provided to caregivers for improvement. By reinforcing nutrition knowledge and promoting better sanitation practices, caregivers were encouraged to gradually reshape their dietary habits and overall household hygiene.

### Anthropometric measurements

To ensure convenience for caregivers, anthropometric measurements were conducted immediately after education sessions at the same locations. With each small group consisting of only 15 to 20 children, we were able to reduce the burden on health workers and minimize errors in data collection. Anthropometric measurements were taken using a weight scale and a standing height scale. For infants and toddlers who could not stand independently, we used length boards and calibrated Salter hanging infant weighing scales. All measurement instruments were carefully calibrated before each session, and every measurement was performed twice to ensure accuracy. If children were unable to attend these sessions, their weight and height data were collected through home visits. To maintain data integrity, all measurements were entered into health workers’ laptops on the same day they were recorded.

### Collection of demographic information

Data were collected using a structured questionnaire through face-to-face interviews conducted by health workers during home visits. The questionnaire was designed to capture the families’ sociodemographic and economic characteristics. The sociodemographic information collected included age, gender, birthdate, number of siblings, birth order, pre-existing medical conditions, primary caregivers (parents, grandparents, or others), minor caregivers, and adherence to vaccination, deworming drugs, and vitamin A supplementation (yes/no). Additional data included whether the family possessed an IDPoor card (yes/no), daily food expenses (<$2.5, $2.5–$5, >$5), the primary food preparer (father, mother, or grandparents), the mother’s education level (none, grade school, middle school, high school, or college), water sources (tap water, well water, fresh/rainwater, or other), and whether drinking water was boiled or filtered (yes/no). The IDPoor card is issued by the Cambodian government as part of its poverty reduction efforts. To qualify, households must complete a standardized questionnaire from the Ministry of Planning. This questionnaire assesses poverty status using a set of proxy indicators, primarily based on observable and verifiable household characteristics such as assets, household size, and education level.

### Statistical methods

Categorical indicators were presented as the number of observations with corresponding proportions. Differences between groups were assessed using the Chi-square test. Continuous variables were expressed as mean and standard deviation, and comparisons between two groups were conducted using Student’s t-test. A two-sided *p*-value of <0.05 was considered statistically significant.

We converted each participant’s actual weight and height into corresponding weight-for-age (WAZ) and height-for-age (HAZ) z-scores using the Nutritional Survey function in WHO Anthro software. Prior to modeling our data, we performed smoothed nonparametric trajectories and ordinary least squares (OLS) regression for preliminary visual graphical analysis. For smoothed nonparametric trajectories, we intentionally categorized participants into three age groups: under 12 months, 12 to 24 months, and over 24 months. However, for OLS analysis, we grouped children into only two categories: under 12 months and over 12 months.

For multi-level modeling (MLM), we constructed two key variables: AGE1 and FOLLOWPERIOD. AGE1 is a binary variable used to classify children based on whether they were younger or older than 12 months at the time of enrollment. Children under 12 months were assigned a value of 0, while those aged 12 months or older were assigned a value of 1. The 12-month cut-off was chosen because it represents a critical period for child growth, during which nutritional interventions may have heightened impact. FOLLOWPERIOD is also a binary variable. Children who enrolled in the GMP during the first phase of implementation were assigned a value of 1, while those who joined in subsequent phases were assigned a value of 0. This variable was created to assess whether earlier participation in the program was associated with improved growth outcomes.

In MLM, a taxonomy of models was established in a sequential order: Model A (unconditional means model), Model B (unconditional growth model), Model C (Model B with the AGE1 variable incorporated), and Model D (Model C with the FOLLOWPERIOD variable incorporated). We compared the values of −2 residual log-likelihood (−2RLL) between consecutive models to assess whether a simpler model should be replaced with a more complex one. An approximate chi-square distribution was used to determine whether the differences in -2RLL between two models were statistically significant.

In model A, one fixed parameter ( $\gamma_{00}$ ), two random parameters ( $\sigma_{0}^{2},\sigma_{\epsilon }^{2}$ ), and intraclass correlation coefficient ρ , defined as $\sigma_{0}^{2}/$ $(\sigma_{0}^{2}+\sigma_{\epsilon }^{2}$ ), were estimated. Model B included two fixed parameters ( $\gamma_{00}$ , $\;\gamma_{01}$ ) and four random variances ( $\sigma_{\varepsilon }^{2},\sigma_{0}^{2},\sigma_{1}^{2}$ , $\sigma_{01}^{2}$ ). Model C extended model B by adding additional two fixed parameters ( $\gamma_{00},\gamma_{01},\gamma_{10},\gamma_{11}$ ) and kept the four random variances ( $\sigma_{\varepsilon }^{2},\sigma_{0}^{2},\sigma_{1}^{2}$ , $\sigma_{01}^{2}$ ). In model D, we included six fixed parameters ( $\;\gamma_{00},\gamma_{01},\gamma_{02},\gamma_{10},\gamma_{11},\gamma_{12}$ ) and the same four random variances ( $\sigma_{\varepsilon }^{2},\sigma_{0}^{2},\sigma_{1}^{2}$ , $\sigma_{01}^{2}$ ).

## Results

A total of 533 children participated in this GMP initially. 175 children eventually dropped out of this program. Finally, a total of 358 children remained in this GMP until the last anthropometric measurement. Of these, 167 children were enrolled in August 2017, 150 joined in April 2018, and an additional 41 entered the program in October 2018. Over the four-year GMP period, a total of 16 anthropometric measurements were conducted. Among the participants, 136 children were 12 months old or younger, while 222 were older than 12 months. Gender distributions between the two age groups were not significantly different. At baseline, children older than 12 months had significantly lower initial height-for-age (HAZ) and weight-for-age (WAZ) z-scores compared to younger children. However, by the end of the program, there were no significant differences in HAZ and WAZ between the two groups. Similarly, the proportions of stunting and wasting were significantly higher in the older age group at the start of the program. However, after 16 measurements, the prevalence of stunting and wasting was no longer significantly different between the two groups (Table 1).

**Table 1:** Baseline characteristics of children with initial and final anthropometric information, Cambodia, 2016–2019.

Characteristics	Whole group (*N* = 358)	Age < 12 months (*N* = 136)	Age ≥ 12 months (*N* = 222)	*p* value
Average age (months, mean ± SD)	17.22 ± 10.39	10.39 ± 6.54	30.32 ± 4.96	
Male (*n* [%])	187 (52.2)	70 (51.5)	117 (52.7)	0.83
Average period of follow-up (months, mean ± SD)	25.57 ± 5.20	23.81 ± 5.40	26.64 ± 4.78	<0.01
Enrolled at the start of GMP	168 (46.9)	45 (33.1)	123 (55.4)	<0.01
HAZ				
Enrolment (mean ± SD)	−1.32 ± 1.32	−0.98 ± 1.48	−1.53 ± 1.17	<0.01
Endpoint (mean ± SD)	−1.18 ± 0.91	−1.09 ± 1.00	−1.24 ± 0.84	0.14
WAZ				
Enrolment (mean ± SD)	−1.21 ± 1.22	−0.86 ± 1.34	−1.44 ± 1.10	<0.01
Endpoint (mean ± SD)	−1.15 ± 0.93	−1.06 ± 1.04	−1.21 ± 0.86	0.19
Stunting				
Enrolment (*n* [%])	90 (26.9)	25 (19.2)	65 (31.7)	0.02
Endpoint (*n* [%])	46 (19.09)	25 (19.0)	37 (18.1)	0.89
Wasting				
Enrolment (*n* [%])	88 (25.4)	23 (17.2)	65 (30.5)	<0.01
Endpoint (*n* [%])	170 (47.5)	27 (19.6)	56 (25.2)	0.30

SD, Standard deviation.

Demographic characteristics of the participants, stratified by age group, are presented in Table 2. Mothers were more likely to serve as the primary caregiver in the younger age group. However, children in this group had lower rates of deworming treatment and vitamin A supplementation. In contrast, older children demonstrated higher rates of vaccine compliance. A larger proportion of families with younger children reported boiling drinking water, although no other significant demographic differences were observed between the groups.

**Table 2:** Demographic characteristics for children and two age groups, Cambodia, 2016–2019.

Characteristics	Whole group (*N* = 358)	Age < 12 months (*N* = 136)	Age > 12 months (*N* = 222)	*p* value
Number of siblings (mean ± SD)	0.82 ± 1.09	0.79 ± 0.98	0.84 ± 1.15	0.64
Main caregiver (Parents, *n* [%])	192 (54.9)	80 (61.1)	112 (51.1)	0.08
IDPoor (*n* [%])	69 (19.3)	22 (16.2)	47 (21.2)	0.27
Vitamin A supplement (*n* [%])	206 (57.5)	59 (43.4)	147 (66.2)	<0.01
De-worming (*n* [%])	141 (40.4)	24 (18.5)	117 (53.4)	<0.01
Vaccination conformity (*n* [%])	247 (70.8)	79 (60.8)	168 (76.7)	<0.01
Mother’s education				0.21
High school completion	132 (37.7)	55 (42.0)	77 (35.2)	
No high school diploma	218 (62.3)	76 (58.0)	142 (64.8)	
Expense on food each day				<0.01
<$2.5 (*n* [%])	87 (24.9)	42 (32.1)	45 (20.6)	
$2.5–$5 (*n* [%])	205 (58.6)	76 (58.0)	129 (58.9)	
>$5 (*n* [%])	58 (16.6)	13 (9.9)	45 (20.6)	
Food preparation				<0.01
Mother	147(42.0)	119(48.0)	28 (27.4)	
Grandparents	178(50.9)	115(46.4)	63(61.8)	
Others	25(7.1)	14(5.7)	11(10.8)	
Water sources				0.054
Tap water	139(39.7)	61(46.6)	78(35.6)	
Others (well/rain/fresh) water	211(60.3)	70(53.4)	141(64.4)	
Boiling water before drinking				0.02
Yes	220 (63.2)	92 (71.3)	128 (58.5)	
No	128 (36.8)	37 (28.7)	91 (41.6)	

**Figure 1:**
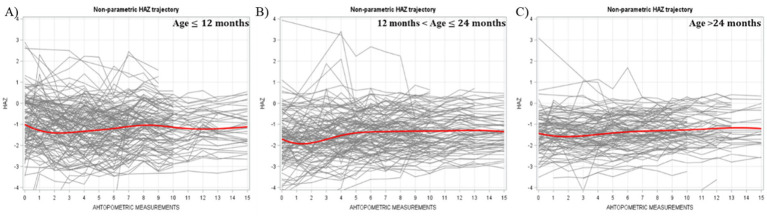
Individual and average HAZ trajectory stratified by age across different measurements (Bold red line represented the average trajectory for the whole group).

Smoothed nonparametric trajectory analyses revealed distinct patterns in HAZ development by age. Children younger than 12 months began with the highest HAZ levels, while those older than 24 months had the lowest. Among the youngest children, HAZ declined initially, reaching its lowest point by the second measurement, before beginning to recover. In children aged 12–24 months, a similar downturn was observed, though the recovery began after the first measurement. For those older than 24 months, HAZ remained relatively stable throughout the study period. These trajectories are illustrated in Figure 1.

**Figure 2:**
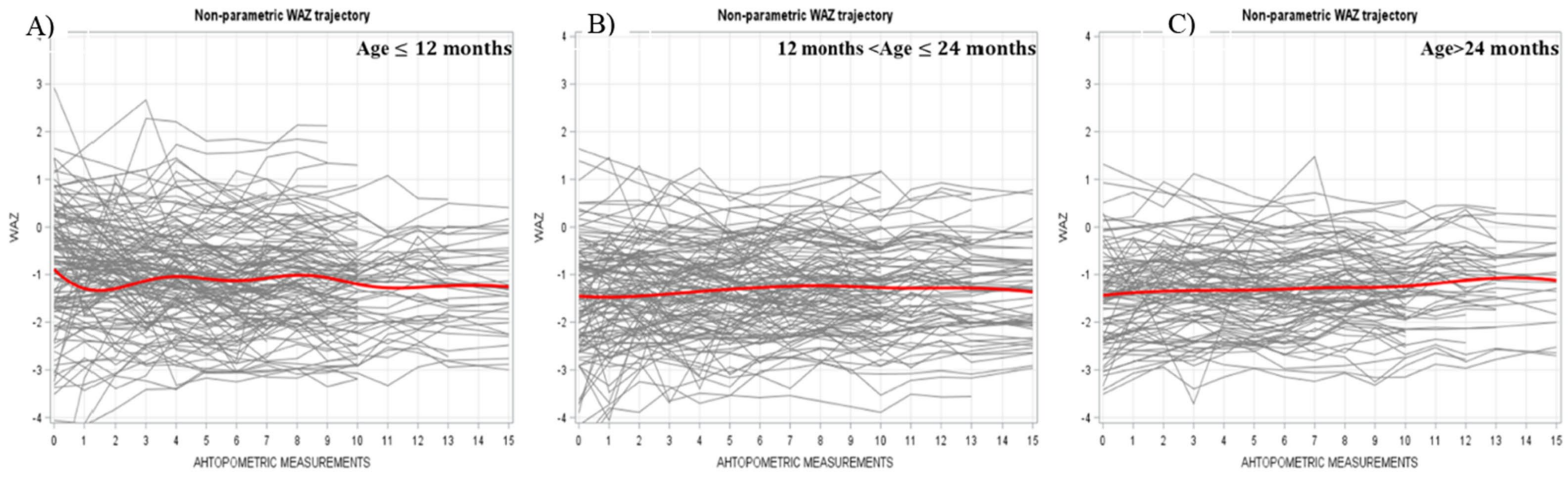
Individual and average WAZ trajectory stratified by age across different measurements (Bold red line represent ted the average trajectory for the whole group).

WAZ trajectories followed a similar pattern in terms of initial levels, with the youngest group starting highest and the oldest group lowest. However, unlike HAZ, the WAZ trajectories for all age groups were relatively flat, showing minimal fluctuation over time. No initial downturns were observed (Figure 2).

### Multi-level modeling of WAZ

**Figure 3:**
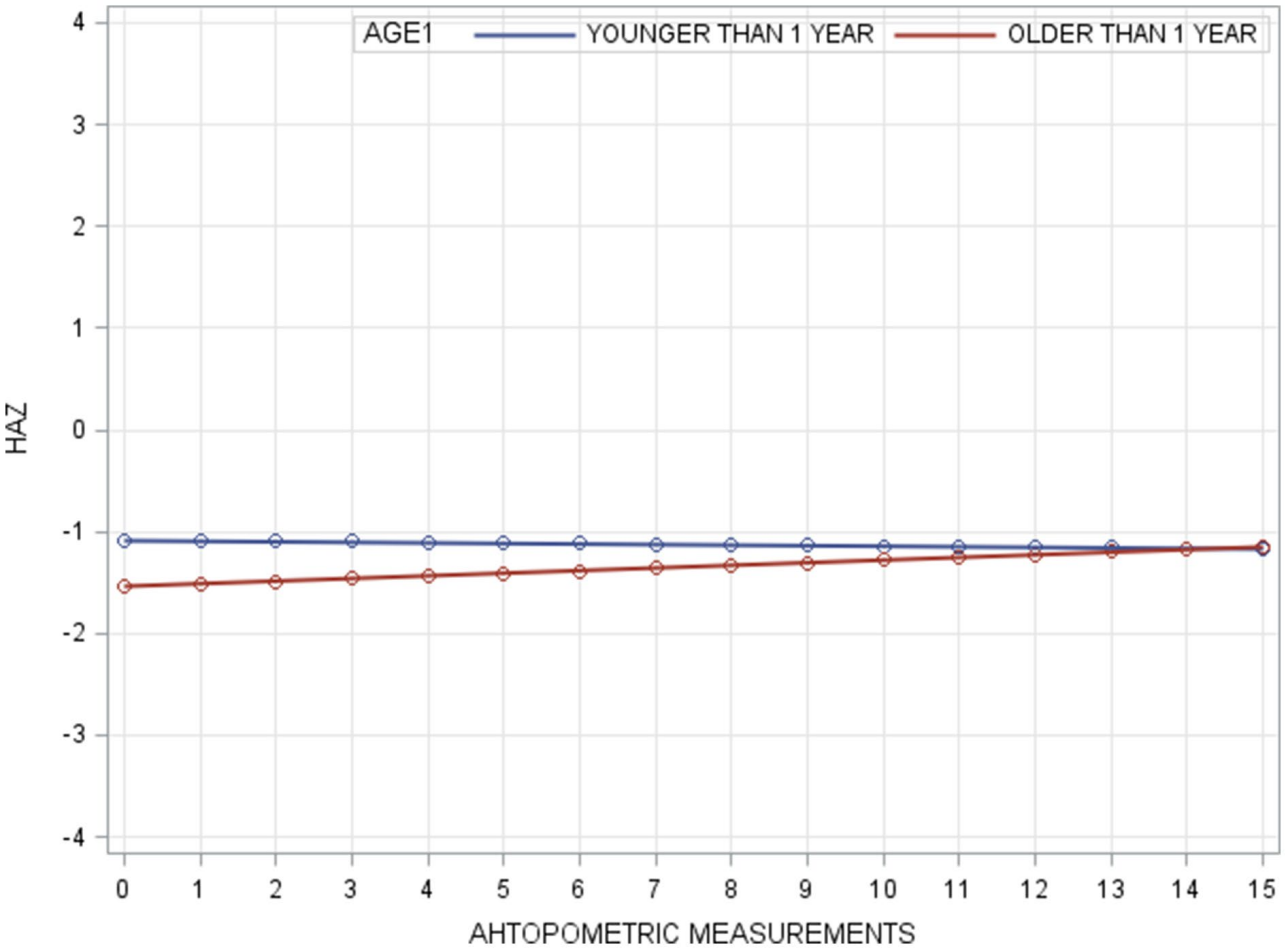
Result of fitted HAZ trajectories for model C.

In the unconditional means model (Model A), the intraclass correlation coefficient (*ρ*) was 0.78, indicating a high proportion of variance in WAZ attributable to between-person differences. Within-person variance (σ^2^_ε_) decreased from 0.21 in Model A to 0.12 in Model B (unconditional growth model), suggesting that 43% of the variance was associated with time (anthropometric measurement points). The improvement in model fit (−2RLL) from Model A to B was significant (*p* < 0.001), justifying the inclusion of the AGE1 variable in Model C. Further addition of the FOLLOWPERIOD variable in Model D did not result in a significant improvement, indicating that while age at enrollment influenced WAZ changes, duration of participation did not. The taxonomy of statistical models for WAZ is shown in Table 3 with detail fixed and random parameters within each model (Figure 3).

**Table 3:** Results of fitting a taxonomy of multilevel models for change to the WAZ data (*n* = 358).

		Parameter	Model A	Model B	Model C	Model D
Fixed effects						
Initial status, π_0i_	Intercept	ϒ_00_ (SE)	−1.19 (0.05)	−1.20 (0.05)	−0.92 (0.09)	−0.87 (0.09)
	AGE1	ϒ_01_ (SE)			−0.45 (0.11)	−0.41 (0.11)
	FOLLOW PERIOD	ϒ_02_ (SE)				−0.17 (0.11)
Rate of change, π_1i_	Intercept	ϒ_10_ (SE)		0.002 (0.004)	−0.02 (0.008)	−0.02 (0.007)
	AGE1	ϒ_11_ (SE)			0.04 (0.01)	0.03 (0.01)
	FOLLOW PERIOD	ϒ_12_ (SE)				0.005 (0.01)
Variance components						
Level 1	Within-person	σ^2^_ε_ (SE)	0.21 (0.005)	0.12 (0.003)	0.12 (0.003)	0.12 (0.003)
Level 2	In initial status	σ^2^_0_ (SE)	0.76 (0.06)	1.00 (0.08)	0.96 (0.08)	0.95 (0.08)
	In rate of change	σ^2^_1_ (SE)		0.007 (0.0005)	0.007 (0.0006)	0.007 (0.0006)
	Covariance	σ^2^_01_ (SE)		−0.04 (0.005)	−0.04 (0.006)	−0.04 (0.005)
Pseudo R^2^ statistics and Goodness-of-fit						
R^2^_YY_			0.004	0.01	0.02	
R_ε_^2^			0.43	0.43	0.43	
R_0_^2^				0.04	0.02	
R_1_^2^				0.00	0.00	
Deviance			6096.8	4918.5	4898.6	4896.1
AIC			6102.8	4930.5	4914.6	4916.1
BIC			6114.4	4953.8	4945.6	4954.9

SE, Standard error.

AGE, dichotomic predictor between age 2 yrs.

FOLLOWPERIOD, dichotomic predictor for participants enrolled in the start of the GMP or not.

WAVE, anthropometric measurements.

### Multi-level modeling of HAZ

**Figure 4:**
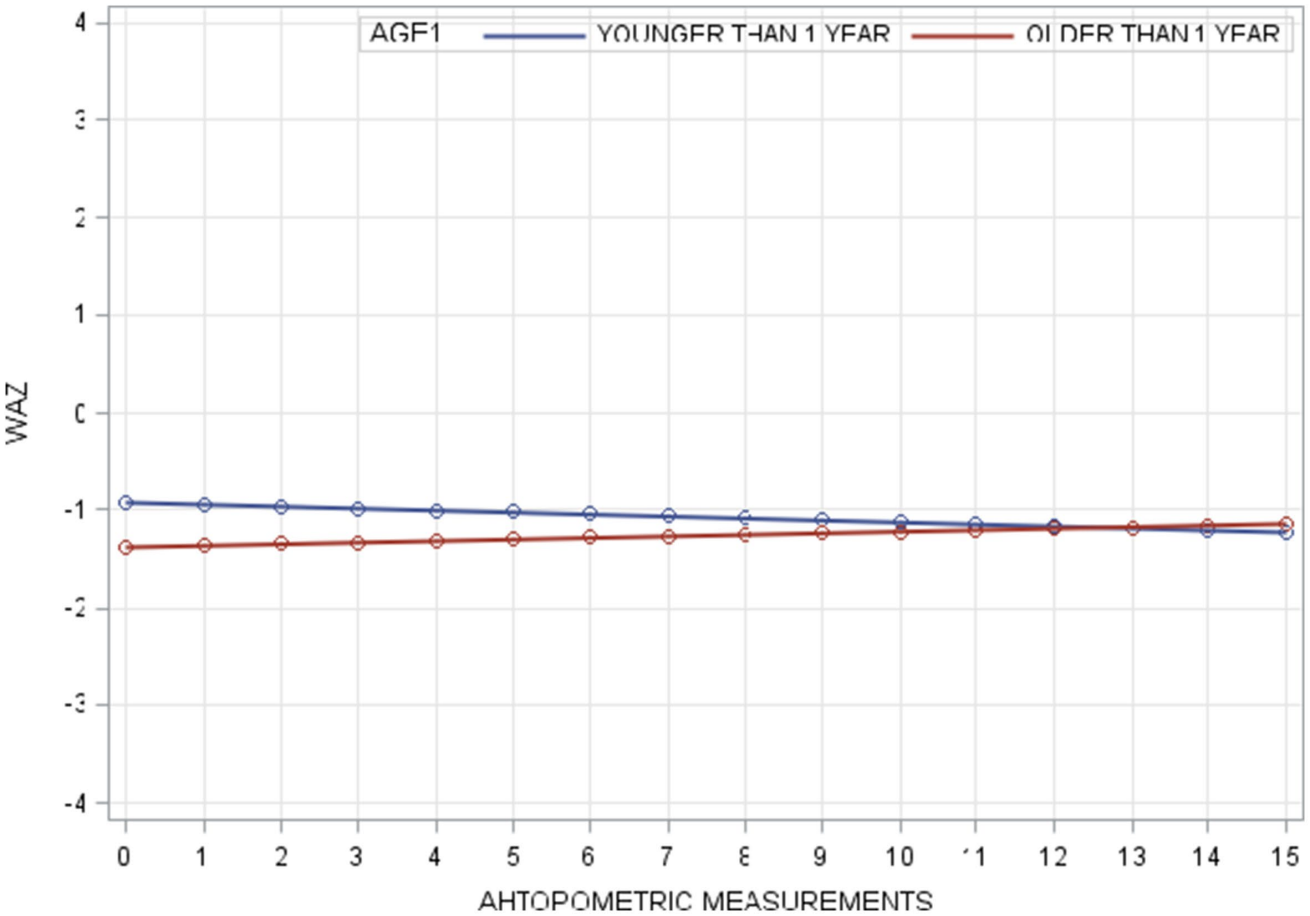
Result of fitted WAZ trajectories for Model C.

For HAZ, Model A yielded an intraclass correlation coefficient of 0.70. Introducing measurement time (Model B) reduced within-person variance from 0.33 to 0.17, accounting for 49% of the variation. The addition of AGE1 in Model C and FOLLOWPERIOD in Model D each significantly improved model fit (all *p* < 0.001), demonstrating that both age at enrollment and length of program participation were significantly associated with HAZ change. The taxonomy of statistical models for HAZ is shown in Table 4 with detail fixed and random parameters within each model (Figure 4).

**Table 4:** Results of fitting a taxonomy of multilevel models for change to the HAZ data (*n* = 358).

		Parameter	Model A	Model B	Model C	Model D
Fixed effects						
Initial status, π_0i_	Intercept	ϒ_00_ (SE)	−1.27 (0.05)	−1.37 (0.06)	−1.08 (0.10)	−0.89 (0.10)
	AGE1	ϒ_01_ (SE)			−0.46 (0.13)	−0.33 (0.13)
	FOLLOW PERIOD	ϒ_02_ (SE)				−0.56 (0.13)
Rate of change, π_1i_	Intercept	ϒ_10_ (SE)		0.01 (0.005)	−0.006 (0.008)	−0.02 (0.009)
	AGE1	ϒ_11_ (SE)			0.03 (0.01)	0.02 (0.01)
	FOLLOW PERIOD	ϒ_12_ (SE)				0.04 (0.01)
Variance components						
Level 1	Within-person	σ^2^_ε_ (SE)	0.33 (0.008)	0.17 (0.004)	0.17 (0.005)	0.17 (0.005)
Level 2	In initial status	σ^2^_0_ (SE)	0.80 (0.06)	1.38 (0.10)	1.34 (0.10)	1.26 (0.10)
	In rate of change	σ^2^_1_ (SE)		0.008 (0.0007)	0.008 (0.0007)	0.008 (0.0007)
	Covariance	σ^2^_01_ (SE)		−0.07 (0.008)	−0.07 (0.007)	−0.06 (0.008)
Pseudo R^2^ statistics and Goodness-of-fit						
R^2^_YY_				0.008	0.02	0.06
R_ε_^2^				0.48	0.48	0.48
R_0_^2^					0.00	0.09
R_1_^2^					0.00	0.00
Deviance			7608.9	6268.0	6254.6	6234.1
AIC			7614.9	6280.0	6044.6	6254.1.7
BIC			7626.5	6303.3	6075.6	6292.9

SE, Standard error.

AGE2, dichotomic predictor between age 2 yrs.

FOLLOWPERIOD, dichotomic predictor for participants enrolled in the start of the GMP or not.

WAVE, anthropometric measurements.

**Figure 5:**
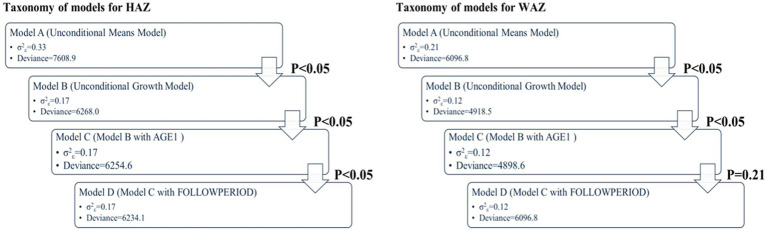
Key results of taxonomy of models for WAZ and HAZ (*p* value: −2RLL difference between two consecutive models using approximate chi-square distribution).

A summary of model specifications and fixed/random effects for WAZ and HAZ is provided in Table 5. Figure 5 illustrates the overall taxonomy of multilevel models used. For WAZ, Model C was selected as the final model since adding FOLLOWPERIOD (Model D) did not significantly improve fit. This implies that while age group influenced WAZ trajectories, the length of participation did not. In contrast, Model D was selected for HAZ, affirming the importance of both age and program duration in explaining HAZ changes.

**Table 5:** Taxonomy of multilevel models for change fitted to the WAZ and HAZ data.

Level 1/Level 2 specification			
Model	Level-1 model	Level-2 model	Composite model
A	Y_ij_ = π_0i_ + ε_ij_	π_0i_ = γ_00_ + ζ_0i_	Y_ij_ = π_0i_ + [γ_00_ + ζ_0i_]
B	Y_ij_ = π_0i_ + π_1i_WAVE_ij_ + ε_ij_	π_0i =_ ϒ_00_ + ζ_0i_ π_1i =_ ϒ_10_ + ζ_1i_	Y_ij_ = ϒ_00_ + ϒ_10_WAVE_ij_ + [ζ_0i_ + ζ_1i_WAVE_ij_ + ε_ij_]
C	Y_ij_ = π_0i_ + π_1i_WAVE_ij_ + ε_ij_	π_0i =_ ϒ_00_ + ϒ_01_AGE1_i_ + ζ_0i_ π_1i =_ ϒ_10_ + ϒ_11_AGE1_i_ + ζ_1i_	Y_ij_ = ϒ_00_ + ϒ_01_AGE1_i_ + ϒ_10_WAVE_ij_ + ϒ_11_AGE1_i_ WAVE_ij_ + [ζ_0i_ + ζ_1i_WAVE_ij_ + ε_ij_]
D	Y_ij_ = π_0i_ + π_1i_WAVE_ij_ + ε_ij_	π_0i =_ ϒ_00_ + ϒ_01_AGE1_i_ + ϒ_02_FOLLOWPERIOD_i_ + ζ_0i_ π_1i =_ ϒ_10_ + ϒ_11_AGE1_i_ + ϒ_12_FOLLOWPERIOD_i_ + ζ_1i_	Y_ij_ = ϒ_00_ + ϒ_01_AGE1_i_ + ϒ_02_FOLLOWPERIOD_i_ + ϒ_10_WAVE_ij_ + ϒ_11_AGE1_i_WAVE_ij_ + ϒ_12_FOLLOWPERIOD_i_ WAVE_ij_ + [ζ_0i_ + ζ_1i_WAVE_ij_ + ε_ij_]

AGE is a dichotomic predictor between age 2 yrs.

FOLLOWPERIOD is a dichotomic predictor for participants enrolled in the start of the GMP or not.

WAVE indicates anthropometric measurements.

## Discussion

The prevalence of stunting and wasting in our study was 26.9 and 25.4%, respectively. While the stunting prevalence aligns with findings from other studies in Cambodia, the prevalence of wasting in our study is notably higher (41). Several past studies have found that the prevalence of wasting among children under 5 years varies across different provinces of Cambodia (5). Given that our GMP was conducted in a rural village in one of the poorest provinces of Cambodia, this higher prevalence of wasting may be attributed to geographic and developmental disparities.

The initial HAZ in the group under 12 months old was higher than that of children above 12 months in our study. This finding aligns with the typical HAZ trajectory observed in developing countries for children from birth to 36 months, where HAZ is highest at birth and declines steadily until reaching its lowest point around 24–36 months. However, the HAZ growth trajectories in our two younger age groups—children under 12 months and those between 12 and 24 months—differed from the expected pattern commonly seen in these settings. Instead of following a continuous decline from birth to 24–36 months, the downward HAZ trajectories in these two younger groups reversed after the second anthropometric measurement. On the other hand, the oldest age group (above 24 months) displayed a relatively flat growth curve, maintaining a stable HAZ level throughout. By the end of our program, the final HAZ values were not significantly different across the three age groups. The WAZ growth trajectories for all three of our groups followed similar patterns to those observed in developing countries, with the curves fluctuating slightly rather than showing a steady decline (42).

We speculate that our GMP may have substantially averted the downward trend of HAZ in the younger groups. A previous study supports this notion, suggesting that a child with a stable HAZ in the first 2 years of life is considered to have better-than-average growth prospects, whereas a stable HAZ beyond 2 years aligns with expected growth patterns (43). Among the three age groups, our program appeared most effective for children under 24 months, as it prevented the decline in HAZ slopes in the two younger groups. Growth faltering during the first 1,000 days of life leads to both short-term and long-term irreversible consequences. Based on our findings, an effective GMP should be implemented before the age of 24 months, as children in this critical period derive the greatest benefits. Early intervention can prevent the physical and neurological consequences associated with undernutrition.

In a longitudinal dataset, the number of measurement waves and their spacing are critical for accurately detecting changes in outcomes. Optimizing the interval between anthropometric measurements presents a challenge in linear growth monitoring, particularly in developing countries. If the interval between measurements is too short, measurement errors may not be minimized. Conversely, if the interval is too long, key characteristics of rapid growth trajectories may be missed (1). The optimal measurement interval is 3–4 months for children under 12 months and 6 months for children older than 12 months (43). Based on this, we designed our GMP with 16 waves of anthropometric measurements spaced every 3 months over 4 years. This frequent and consistent measurement schedule allows for a more detailed and robust statistical model.

Before proceeding to model-based analysis, exploratory graphical analyses provide preliminary insights into the characteristics of our data. A key advantage of smoothed nonparametric trajectory is that it does not require strict assumptions and can effectively capture temporal changes of interest. Nonparametric graphical analysis also offers guidance in selecting an appropriate functional form for the next stage of parametric analysis (44). For instance, in our study, the two younger age groups exhibited a downward HAZ trajectory spanning only 3–6 months. If we had initially specified a parametric growth model without exploratory visualization, we might have overlooked these short-term declines in HAZ. However, while nonparametric models are useful for visualizing trends, they do not generate interpretable parameters for hypothesis testing or generalization. Therefore, after the initial visual analysis, a parametric model is necessary to quantify effects and draw meaningful conclusions.

With the aim of addressing undernutrition issues in Africa, growth monitoring (GM) emerged in the 1960s (45). However, early stand-alone GM programs that focused solely on regular weight and height measurements had minimal impact on improving child undernutrition. Over time, follow-up interventions were integrated into traditional GM, evolving into what is now known as growth monitoring and promotion (GMP). In practice, the interventions included in a GMP are tailored to the specific needs of different communities. Ideally, the scope of interventions should align with local nutritional contexts, but financial constraints and public health infrastructure often limit the extent of implementation. As a result, GMP programs typically encompass a bundle of interventions, making it difficult to determine which specific elements drive positive outcomes. Through a literature review, we observed that the effectiveness of GMP programs depends on multiple factors. Guided by past successes and failures, we designed a comprehensive and sustainable GMP. Our target population included children aged 6 months to 5 years, with a focus on enrolling children under 24 months old, as this is a critical period for child development. Since we did not have a control group, we stratified our study population into two groups for modeling: children under 12 months and those above 12 months old. This stratification formed the foundation of our analysis. The results of our study provided an answer to our research question, demonstrating that our GMP effectively modified the typical HAZ trajectory observed in children under 24 months within this community. We were unable to find publications investigating whether the duration of a GMP influences its success. Most GMPs typically last between 6 months and 2 years. In our study, we deliberately extended the program to 4 years to assess the potential benefits of a longer intervention period. Our study included three groups of children who entered the GMP at different time points. Based on the results of Model D, we observed that the longer a child remained in the program, the greater the improvement in HAZ. However, we did not find a significant impact of follow-up duration on WAZ trajectory. This aligns with existing evidence that HAZ is a more sensitive indicator of chronic undernutrition compared to WAZ. Given these findings, we believe that extending the duration of GMPs may have a more substantial impact on reducing chronic undernutrition in children in developing countries.

Workforce capacity influences the efficacy of a GMP delivery directly. A workforce mixed with paid, trained workers and community volunteers has a synergistic effect in delivering GMP interventions (46, 47). A trained worker can deliver in-depth nutrition counseling and accurate anthropometric measurements. Local volunteers can help cultivate connections between community workers and local families. Optimization of workload is another issue that influences the efficacy of a GMP. In our GMP, less than 100 children were assigned to each community worker. A realistic ratio between workers and families guarantees the quality of nutritional counseling during home visits. Besides, a full-time pediatrician provided supportive supervision for our community workers and helped resolve challenges community workers faced during home visits. The supportive supervision of community-level workers from the next level of healthcare worker ensured the consistency of intervention delivery.

The effectiveness of our GMP was also influenced by several external factors. Past studies have identified a lack of caregiver participation, poor understanding of growth monitoring concepts, and weak community involvement as key barriers to success (48). To address these challenges, we strategically located our anthropometric measurement sites and nutrition education sessions close to caregivers’ residences. Additionally, we conducted these sessions in small group settings, which facilitated bi-directional communication between community workers and caregivers. This approach not only enhanced engagement but also fostered a positive deviance effect, wherein caregivers could learn from those who successfully applied best practices in child nutrition. Positive deviance approach has been proven effective in community-based programs (49). By implementing these strategies, we aimed to improve caregiver participation and community involvement, thereby strengthening the overall impact of our GMP.

Community-based monitoring alone, without home visits, has been shown to have a limited impact on child nutrition (50). In our GMP, home visits were a cornerstone of the intervention strategy, providing a direct platform for follow-up actions. These visits allowed us to reinforce proper child-feeding practices, monitor progress, and ensure that caregivers received personalized guidance. A GMP is unlikely to succeed if mothers lack awareness of appropriate feeding practices or if they do not receive support from other family members (51). Home visits helped foster family-wide consensus on nutrition practices, increasing the likelihood of long-term adherence. Another crucial factor influencing GMP success is the mother’s education level. Studies have consistently shown that higher maternal education correlates with better child nutrition outcomes (52). In this region, we found that many caregivers were grandparents. Hence, individual tailored nutrition counseling can compensate for the knowledge gap of caregivers. At the community level, we established a partnership with local stakeholders through monthly meetings. These meetings ensured that local leaders, health workers, and policymakers were actively involved in shaping and implementing our GMP interventions. Engaging local stakeholders in the policy design process from the early stages allowed us to tailor interventions to the specific needs of the community. This collaboration not only improved program relevance and sustainability but also enhanced community buy-in, increasing participation and adherence to GMP recommendations.

There were several limitations in our study that must be acknowledged. First, the GMP was implemented on a small scale, involving only 368 children, and was geographically limited to a single village. As a result, the findings may not be directly applicable to other communities or settings. Selection bias may also be present, as parents who chose to participate in the program might have been more motivated to improve their children’s nutrition, potentially influencing the study’s outcomes.

Another limitation is that children entered the GMP at different ages, placing them at varying stages of their growth trajectories. For instance, some children might have already reached the nadir of their height-for-age z-score (HAZ) and begun to improve, while others were still experiencing growth declines. This heterogeneity in growth curves within the same age group may not accurately represent the average HAZ trajectories of all individuals in that group.

Recommendations for future studies include enrolling a larger number of children and dividing them into narrower age groups to reduce the variability in growth trajectories. This approach would allow for more precise analysis. Additionally, quantifying changes in caregiver behavior could help establish a clearer causal link between program interventions, behavioral changes, and improvements in child nutrition.

In conclusion, we believe that an effective GMP hinges on frequent and bidirectional communication between caregivers and community workers. This dialogue must be tailored to the unique needs of individual households and the broader community. Moreover, a longer GMP duration appears to provide more significant benefits for combating child undernutrition than a shorter program duration.

## Data Availability

The raw data supporting the conclusions of this article will be made available by the authors, without undue reservation.
